# Supplementary material for: Dry reforming of methane over gallium-based supported catalytically active liquid metal solutions
Source: Commun Chem. 2023 Oct 18;6:224. doi: 10.1038/s42004-023-01018-w (PMC10584823; doi:10.1038/s42004-023-01018-w)
Supplement: Supplementary file 2 — Supplementary Information [file 42004_2023_1018_MOESM2_ESM.pdf]

Supporting information:

Dry reforming of methane over gallium-based supported catalytically active liquid metal solutions

Moritz Wolf,[1,2] Ana Luiza de Oliveira,[1,2] Nicola Taccardi,[1] Sven Maisel,[3] Martina Heller,[4] Sharmin Khan Antara,[1] Alexander Søgaard,[1] Peter Felfer,[4] Andreas Görling,[3] Marco Haumann,[1] Peter Wasserscheid*,[1,2]

1) Friedrich-Alexander-Universität Erlangen-Nürnberg (FAU), Lehrstuhl für Chemische Reaktionstechnik (CRT), Egerlandstr. 3, 91058 Erlangen, Germany.

2) Forschungszentrum Jülich, Helmholtz Institute Erlangen-Nürnberg for Renewable Energy (IEK 11), Cauerstr. 1, 91058 Erlangen, Germany.

3) Friedrich-Alexander-Universität Erlangen-Nürnberg (FAU), Lehrstuhl für Theoretische Chemie, Egerlandstr. 3, 91058 Erlangen, Germany.

4) Friedrich-Alexander-Universität Erlangen-Nürnberg (FAU), Lehrstuhl für Werkstoffwissenschaften (Allgemeine Werkstoffeigenschaften), Martensstr. 5, 91058 Erlangen, Germany.

*Corresponding author. E-mail address: peter.wasserscheid@fau.de

Supporting figures

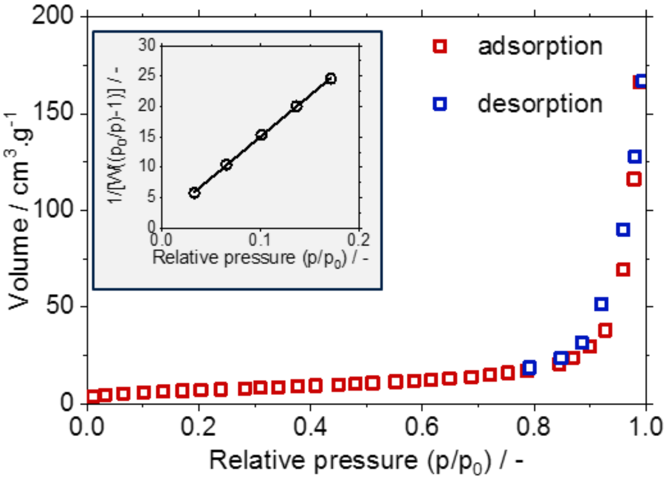

Figure S1: Nitrogen adsorption-desorption isotherm with linear fit to the Brunauer-Emmett-Teller equation (inset) of the utilised β-SiC support material.

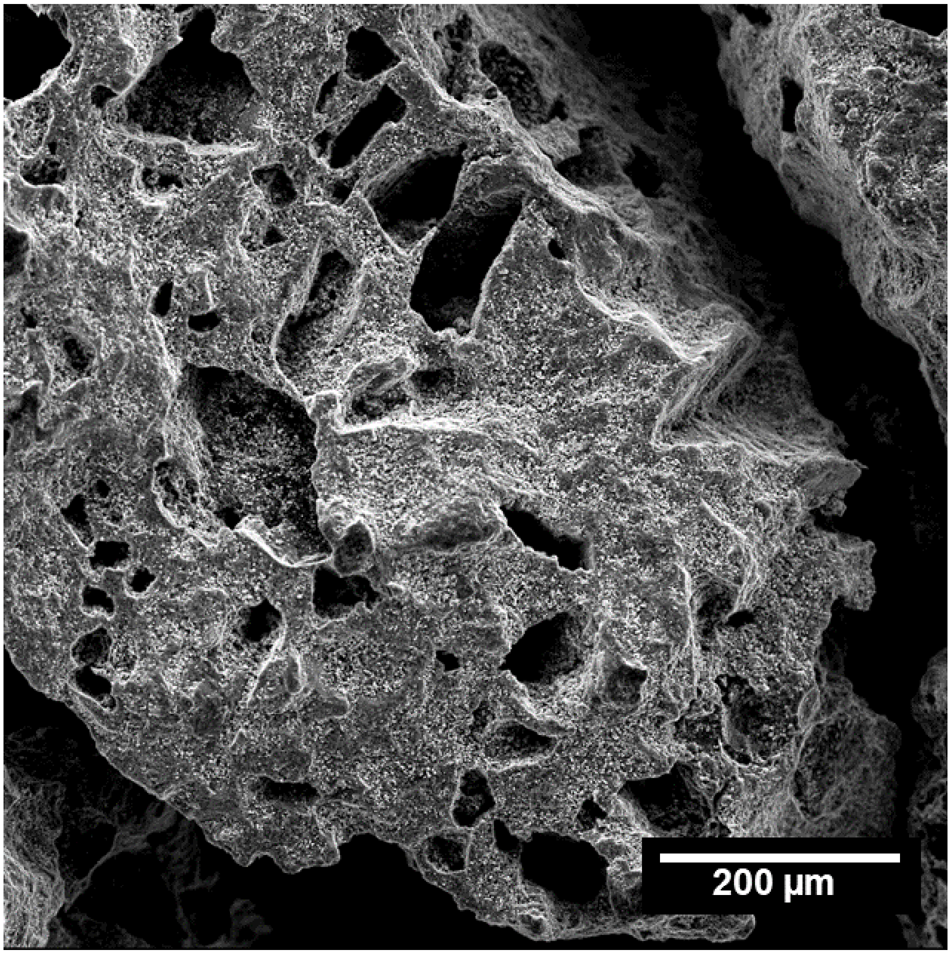

Figure S2: Secondary electron scanning electron micrograph of the mesoporous β-SiC support.

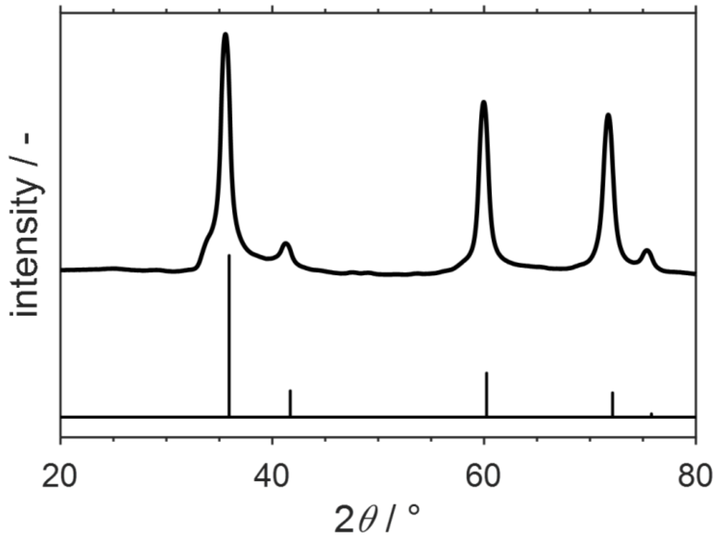

Figure S3: X-ray diffractogram (Cu K-alpha radiation with λ = 1.541 Å) of the mesoporous β-SiC support with a reference pattern.

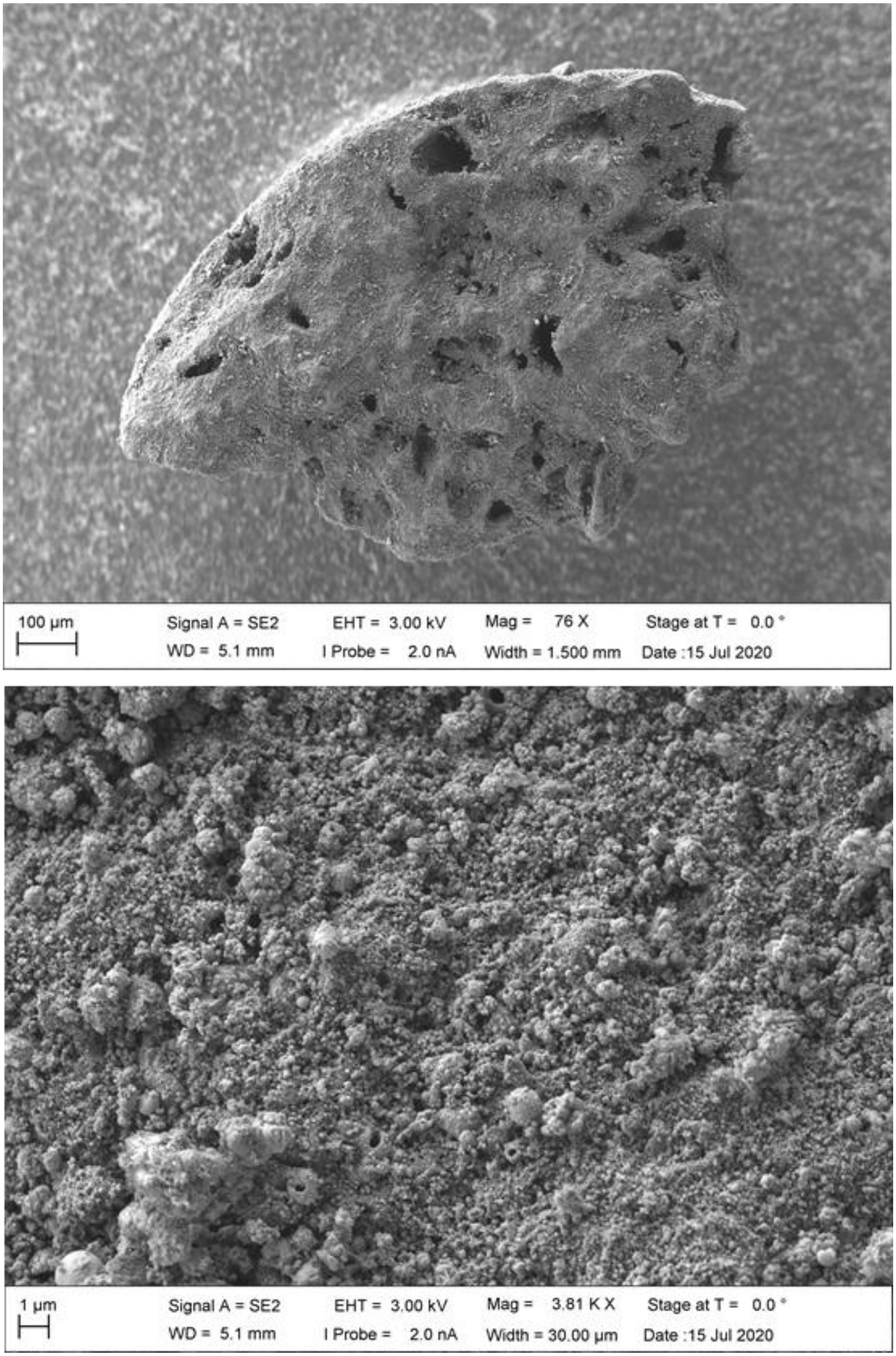

Figure S4: Scanning electron micrographs of $Ga_{45}Ni/SiC$ with metal loadings of Ga and Ni of 4.52 and 0.08 wt.%, respectively.

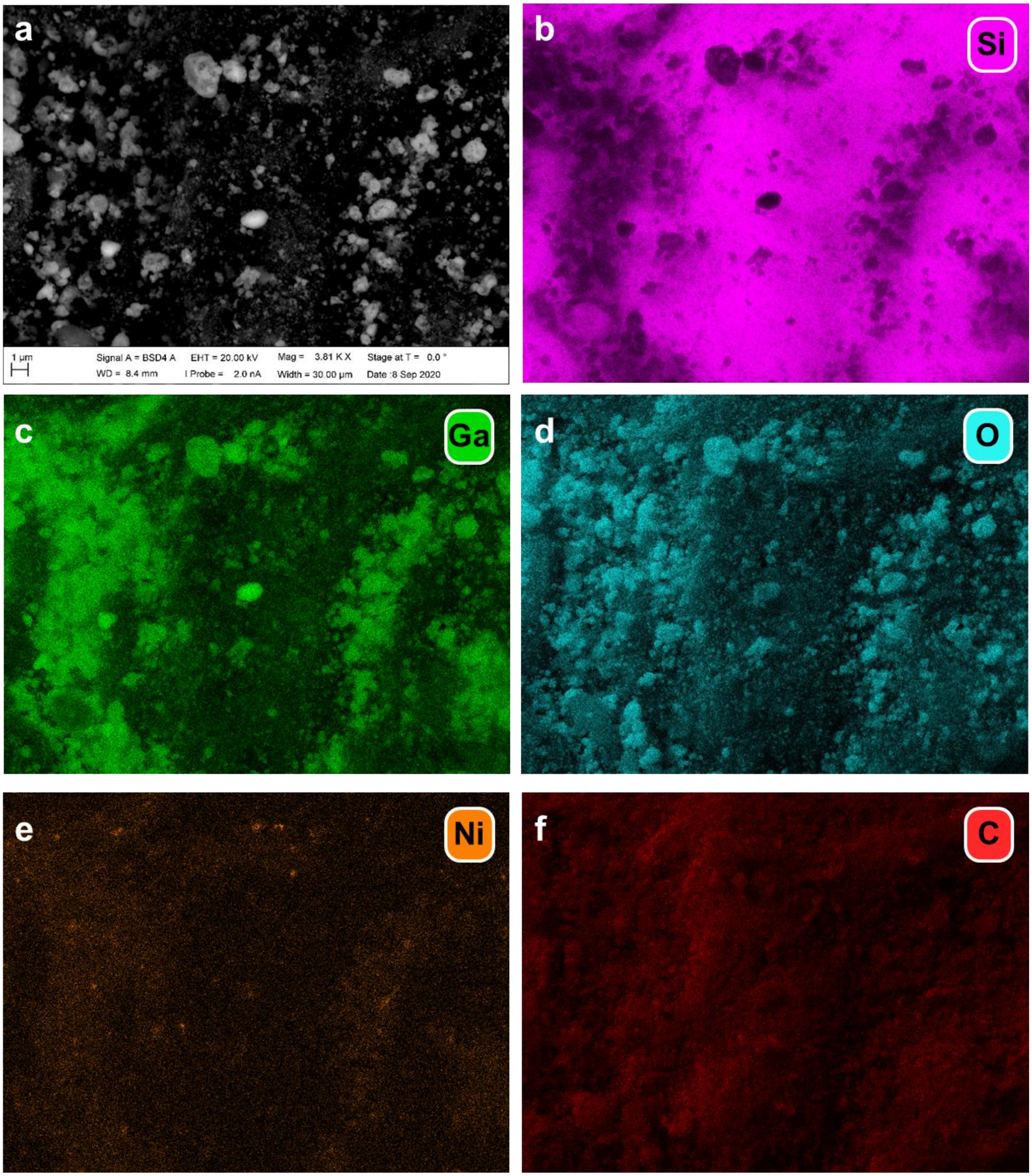

Figure S5: (a) Backscattered scanning electron micrograph with the corresponding (b-f) elemental maps via energy-dispersive X-ray spectroscopy of $Ga_{45}Ni/SiC$ with metal loadings of Ga and Ni of 4.52 and 0.08 wt.%, respectively.

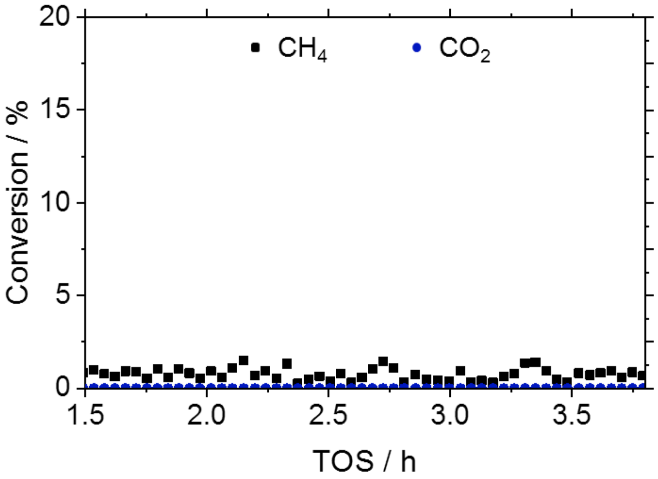

Figure S6: Conversion of CH_4 and CO_2 in the blank quartz tube reactor. Reaction conditions: 900 °C, 1 bar, CH_4:CO_2:Ar = 1:1:3, 50 mL_N min^{-1}.

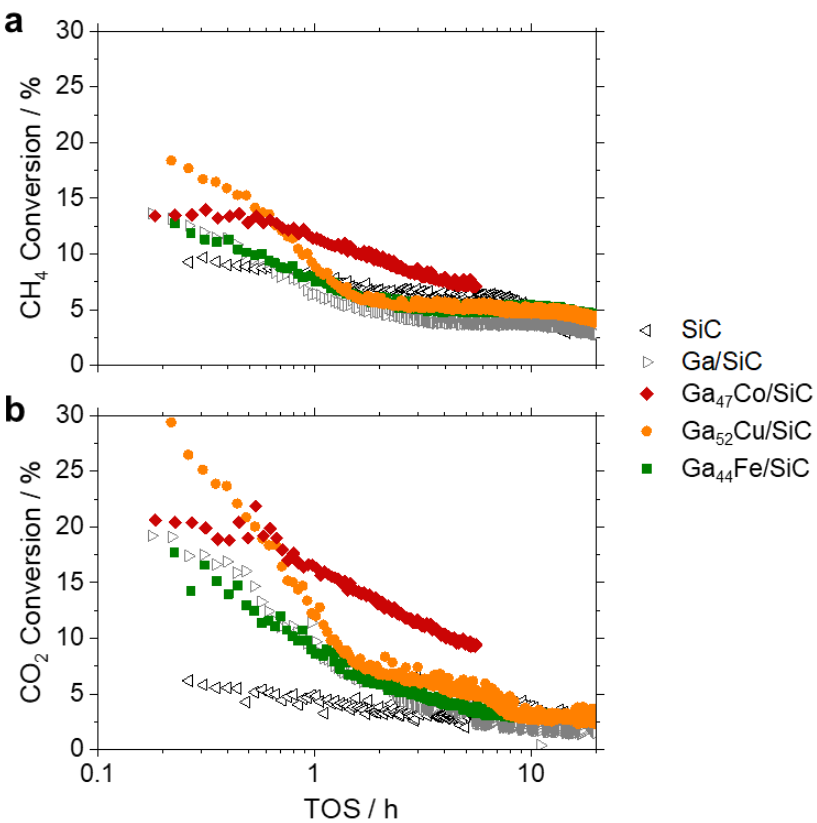

Figure S7: Conversion of (a) CH_4 and (b) CO_2 during dry reforming of methane over Ga-based SCALMS with various active metals employing a mesoporous SiC support, as well as a Ga/SiC and bare SiC reference samples. Metal loadings: 5.50 wt.% Ga for Ga/SiC, 4.85 wt.% Ga and 0.09 wt.% Co for $Ga_{47}Co$/SiC, 4.91 wt.% Ga and 0.09 wt.% Cu for $Ga_{52}Cu$/SiC, 4.53 wt.% Ga and 0.08 wt.% Fe for $Ga_{44}Fe$/SiC. Reaction conditions: 900 °C, 1 bar, 1 g catalyst, CH_4:CO_2:Ar = 1:1:3, 3 L_N g_{cat}^{-1} h^{-1}.

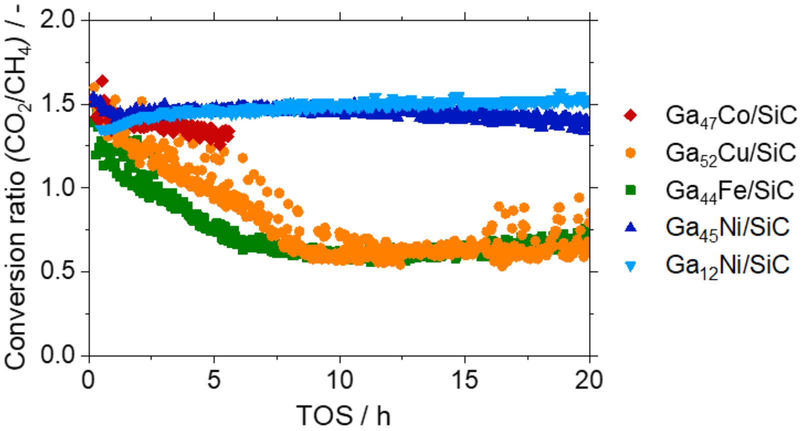

Figure S8: Ratio of the conversion of CO_2 and CH_4 during dry reforming of methane over Ga-based SCALMS with various active metals employing a mesoporous SiC support, as well as a Ga/SiC and bare SiC reference samples. Metal loadings: 4.85 wt.% Ga and 0.09 wt.% Co for $Ga_{47}Co/SiC$, 4.91 wt.% Ga and 0.09 wt.% Cu for $Ga_{52}Cu/SiC$, 4.53 wt.% Ga and 0.08 wt.% Fe for $Ga_{44}Fe/SiC$, 4.52 wt.% Ga and 0.08 wt.% Ni for $Ga_{45}Ni/SiC$, 5.44 wt.% Ga and 0.38 wt.% Ni for $Ga_{12}Ni/SiC$. Reaction conditions: 900 °C, 1 bar, 1 g catalyst, CH_4:CO_2:Ar = 1:1:3, 3 L_N g_{cat}^{-1} h^{-1}.

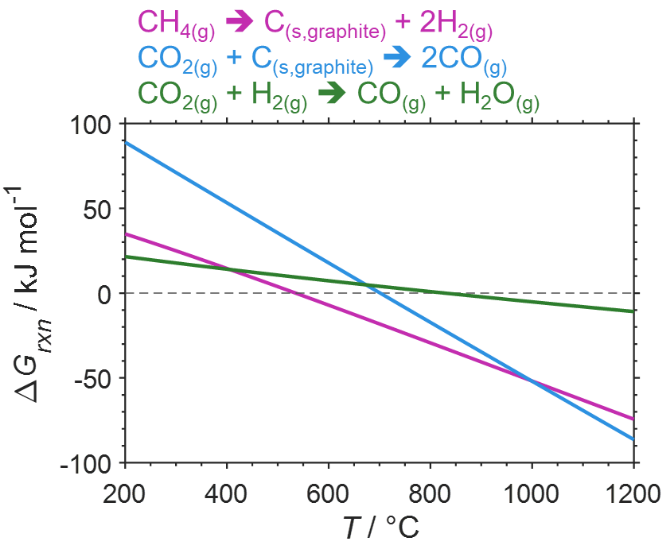

Figure S9: Gibbs free energies for methane activation, as well as the Boudouard reaction and the reverse water gas shift. Calculations are based on thermodynamic data from Knacke et al.[1-3]

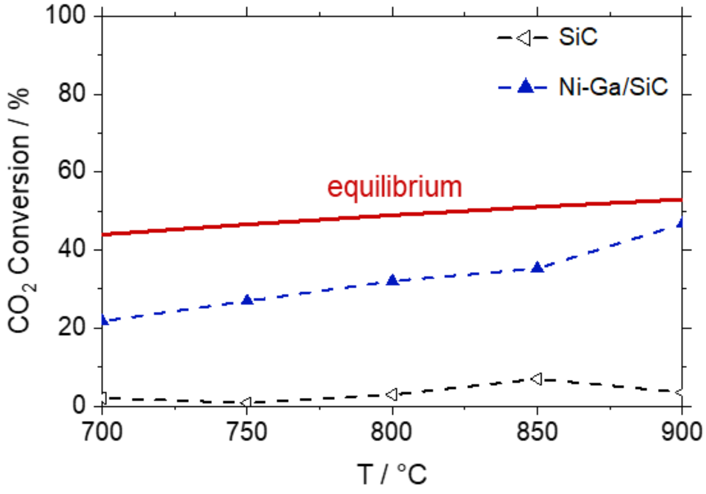

Figure S10: Conversion of CO_2 and H_2 over $Ga_{45}Ni/SiC$ and the bare SiC support material for evaluation of the potential conversion of CO_2 via the reverse water gas shift reaction during dry reforming of methane. Metal loading: 4.52 wt.% Ga and 0.08 wt.% Ni for $Ga_{45}Ni/SiC$. Reaction conditions: 700-900 °C, 1 bar, 1 g catalyst, $H_2:CO_2:Ar$ = 1:1:3, 3 L_N g_{cat}^{-1} h^{-1}.

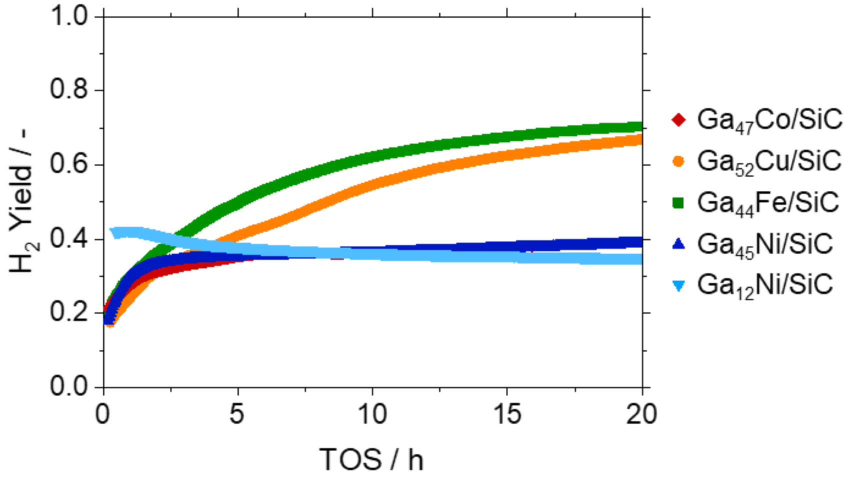

Figure S11: Yield of H_2 from converted CH_4 during dry reforming of methane over Ga-based SCALMS with various active metals employing a mesoporous SiC support. Metal loadings: 4.85 wt.% Ga and 0.09 wt.% Co for $Ga_{47}Co/SiC$, 4.91 wt.% Ga and 0.09 wt.% Cu for $Ga_{52}Cu/SiC$, 4.53 wt.% Ga and 0.08 wt.% Fe for $Ga_{44}Fe/SiC$, 4.52 wt.% Ga and 0.08 wt.% Ni for $Ga_{45}Ni/SiC$, 5.44 wt.% Ga and 0.38 wt.% Ni for $Ga_{12}Ni/SiC$. Reaction conditions: 900 °C, 1 bar, 1 g catalyst, $CH_4:CO_2:Ar$ = 1:1:3, 3 L_N g_{cat}^{-1} h^{-1}.

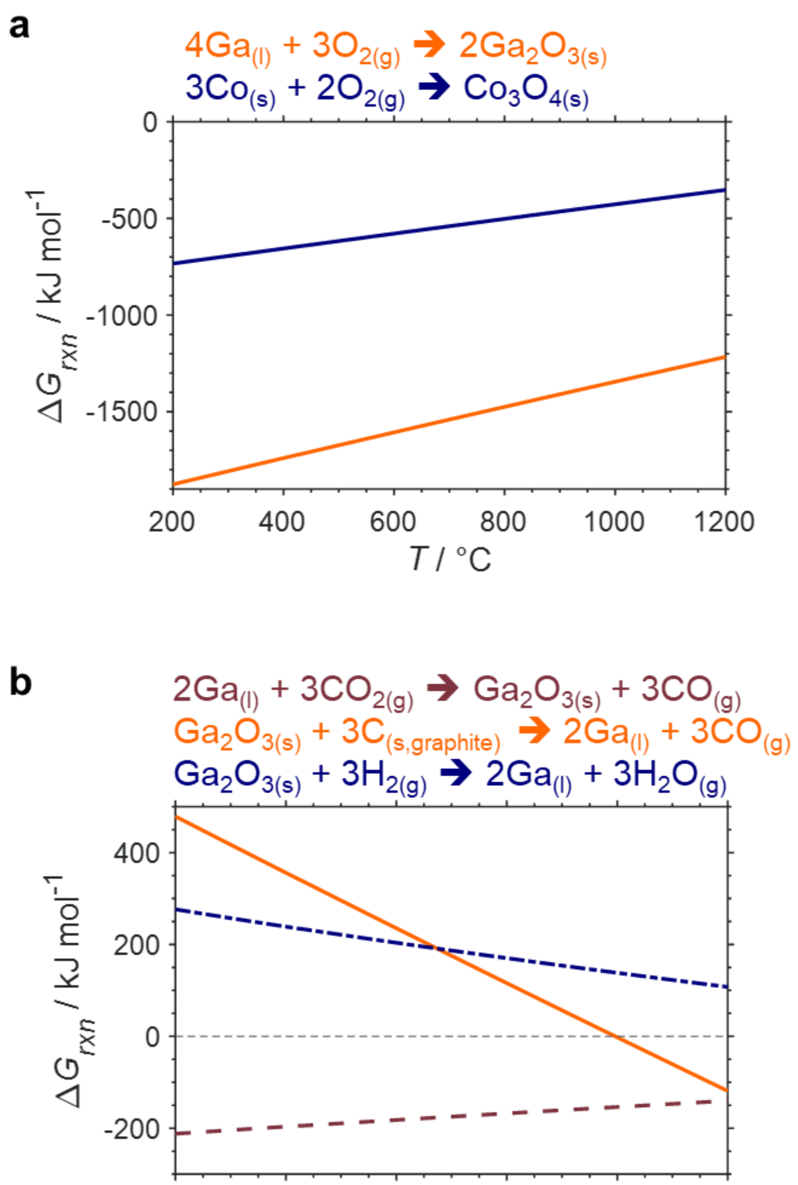

Figure S12: Gibbs free energies for (a) the oxidation of liquid gallium and solid cobalt by oxygen forming gallium(III) oxide and cobalt(II,III) oxide, respectively, and (b) the oxidation of liquid gallium by carbon dioxide and the reduction of gallium oxide by graphitic carbon or hydrogen. Calculations are based on thermodynamic data from Knacke et al.[1-3]

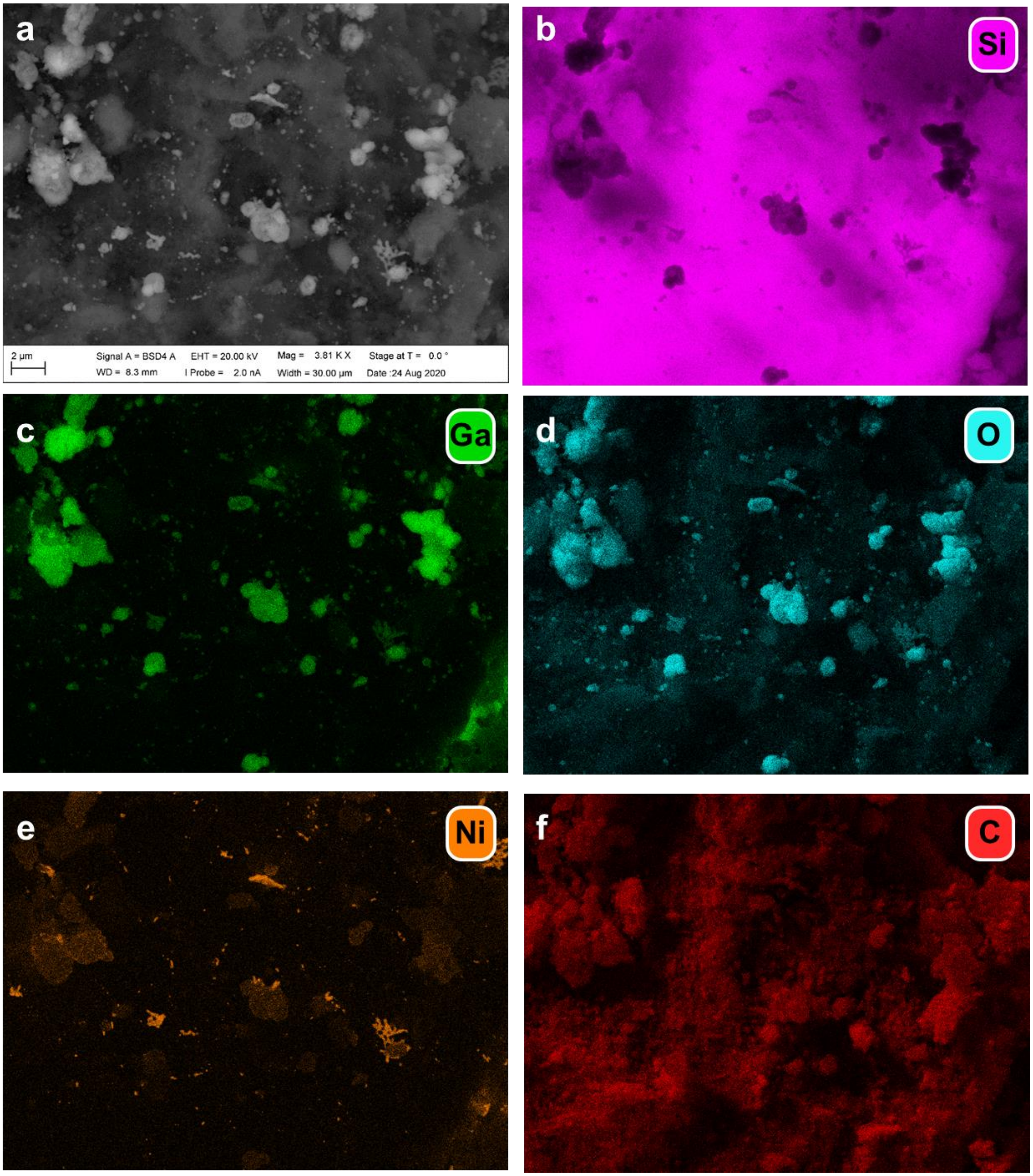

Figure S13: (a) Backscattered scanning electron micrograph with the corresponding (b-f) elemental maps via energy-dispersive X-ray spectroscopy of $Ga_{12}Ni/SiC$ with metal loadings of Ga and Ni of 5.44 and 0.38 wt.%, respectively.

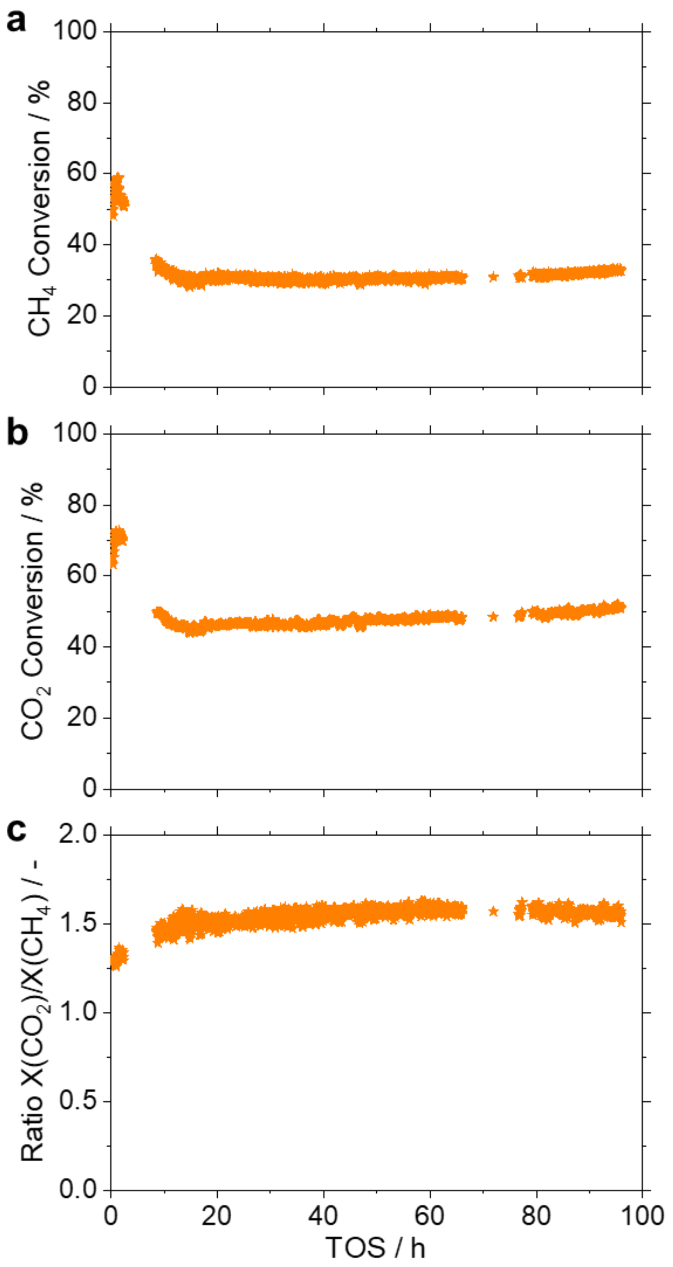

Figure S14: Conversion of (a) CH_4 and (b) CO_2, as well as (c) the ratio of the conversion of CO_2 over CH_4 during dry reforming of methane over $Ga_{12}Ni/SiC$ SCALMS during a long-term experiment over 100 h. Metal loadings: 5.44 wt.% Ga and 0.38 wt.% Ni. Reaction conditions: 900 °C, 1 bar, 1 g SCALMS, CH_4:CO_2:Ar = 1:1:3, 3 L_N g_{cat}^{-1} h^{-1}.

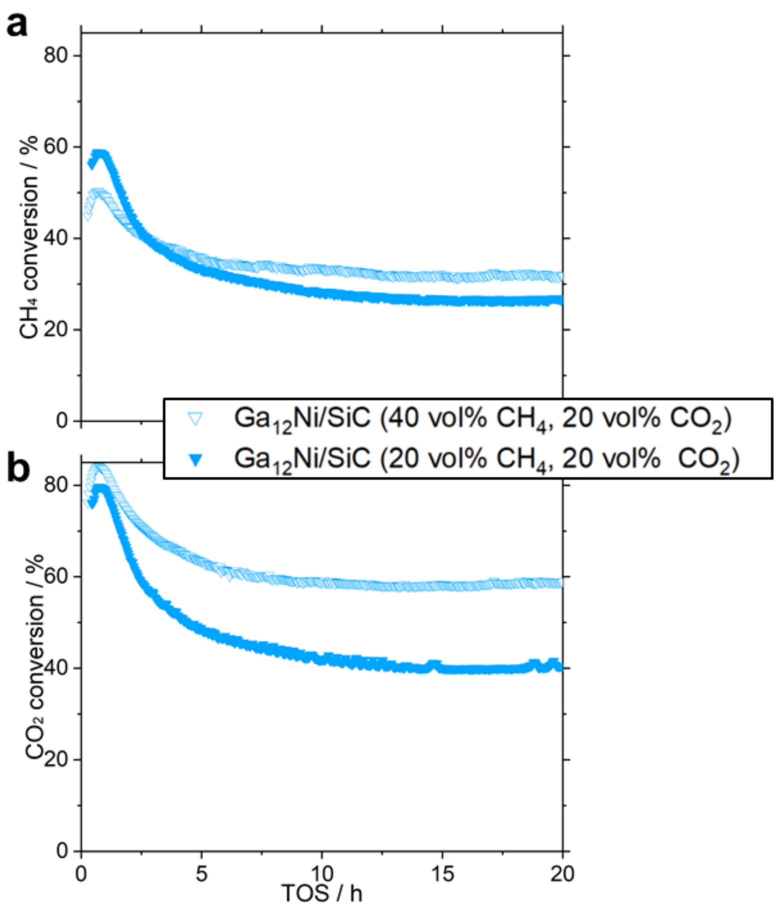

Figure S15: Conversion of (a) CH_4 and (b) CO_2 during dry reforming of methane over $Ga_{12}Ni$/SiC SCALMS with either 40% or 20% CH_4 in the feed stream. Metal loadings: 5.44 wt.% Ga and 0.38 wt.% Ni. Reaction conditions: 900 °C, 1 bar, 1 g SCALMS, CH_4:CO_2:Ar = 1:1:3 respectively 2:1:2, 3 L_N g_{cat}^{-1} h^{-1}.

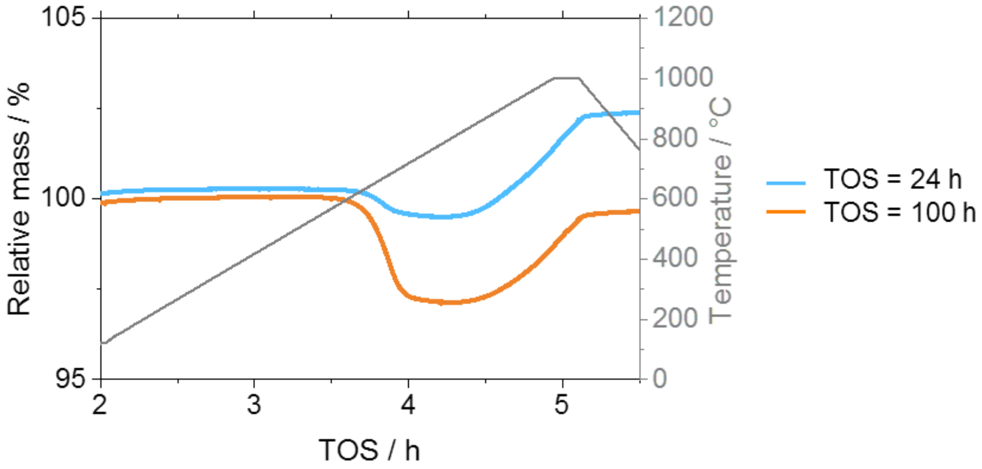

Figure S16: Thermogravimetric analysis during temperature-programmed oxidation of the spent $Ga_{12}Ni$/SiC SCALMS after 24 h time on stream and after 100 h long-term application in dry reforming of methane.

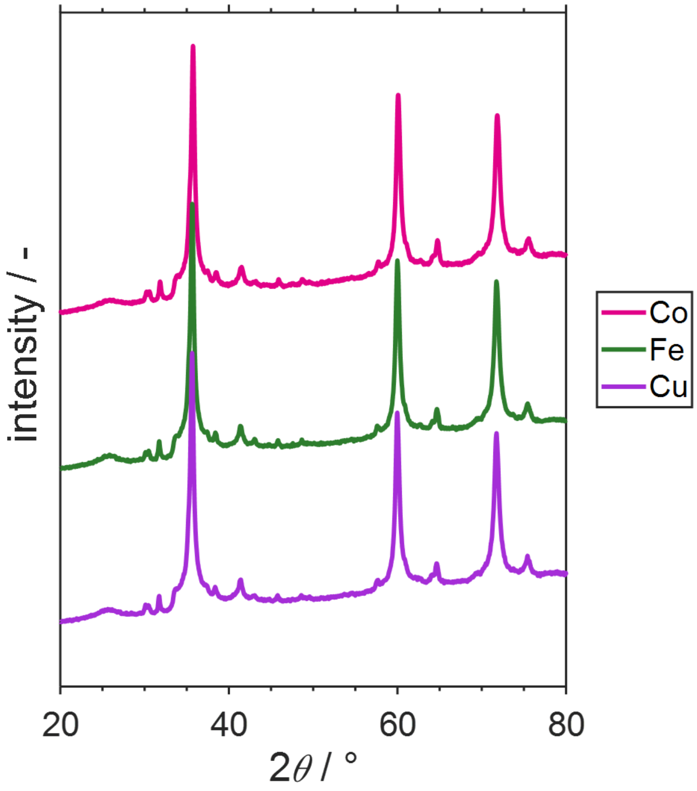

Figure S17: X-ray diffractograms (Cu K-alpha radiation with λ = 1.541 Å) of Ga-Co, Ga-Fe, and Ga-Cu SCALMS employing a mesoporous SiC support after application in dry reforming of methane.

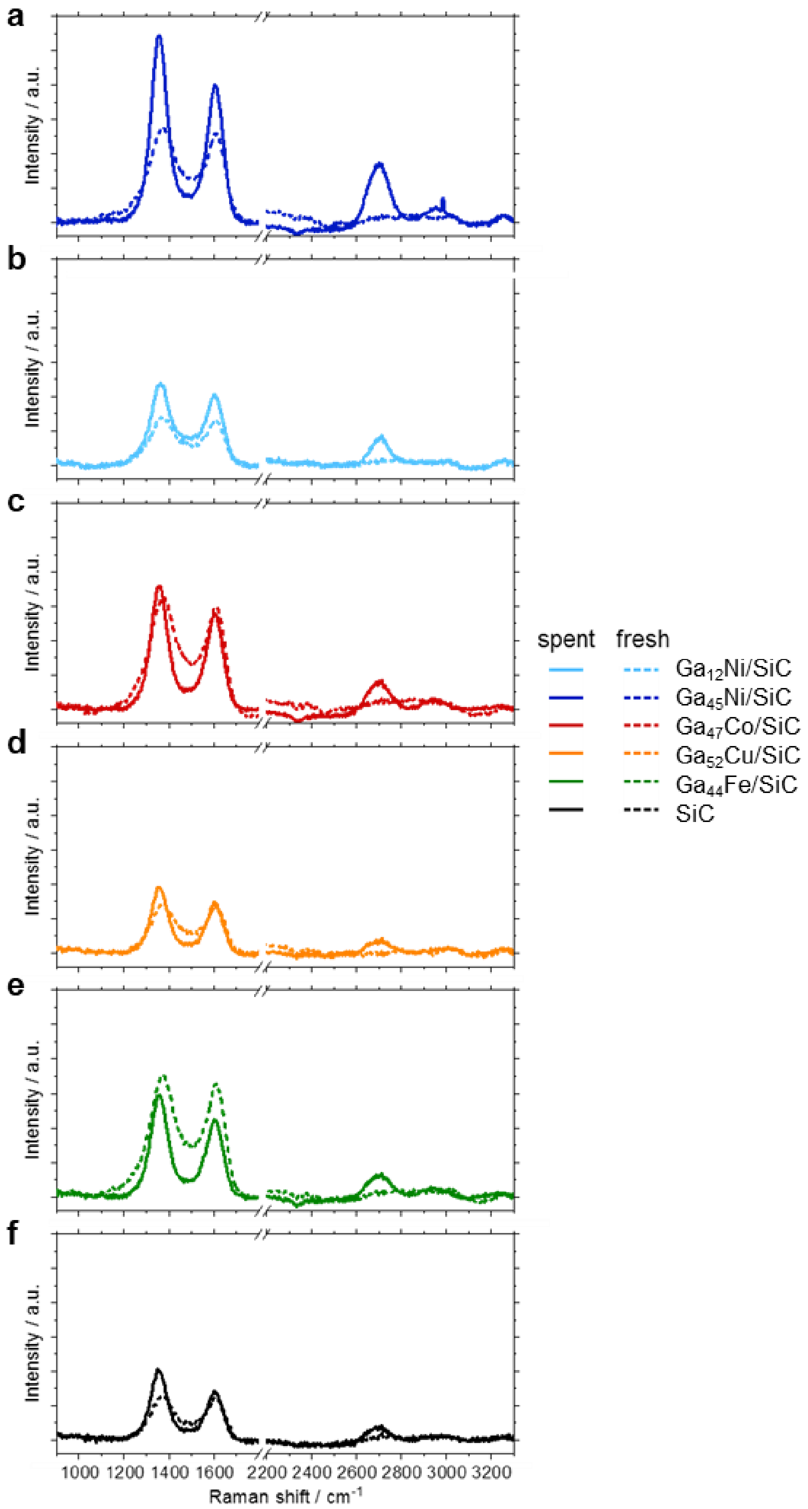

Figure S18: Raman spectra of (a-e) SCALMS with various active metals employing a mesoporous SiC support, as well as (f) the bare support material before and after application in dry reforming of methane. Metal loadings: 4.85 wt.% Ga and 0.09 wt.% Co for $Ga_{47}Co/SiC$, 4.91 wt.% Ga and 0.09 wt.% Cu for $Ga_{52}Cu/SiC$, 4.53 wt.% Ga and 0.08 wt.% Fe for $Ga_{44}Fe/SiC$, 4.52 wt.% Ga and 0.08 wt.% Ni for $Ga_{45}Ni/SiC$, 5.44 wt.% Ga and 0.38 wt.% Ni for $Ga_{12}Ni/SiC$.

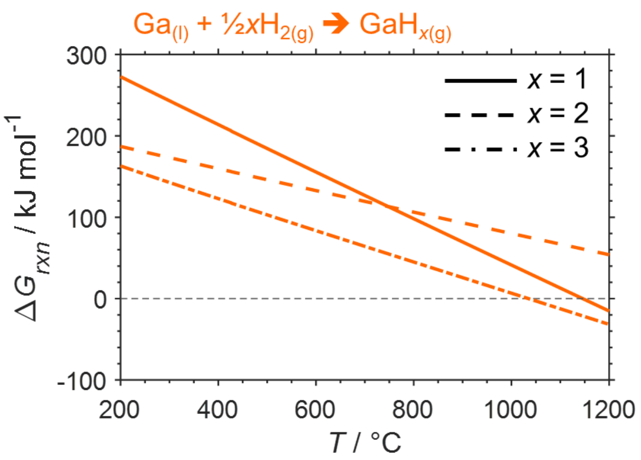

Figure S19: Gibbs free energy for the formation of gallium hydrides from liquid gallium. Calculations are based on thermodynamic data from Knacke et al.[1-3]

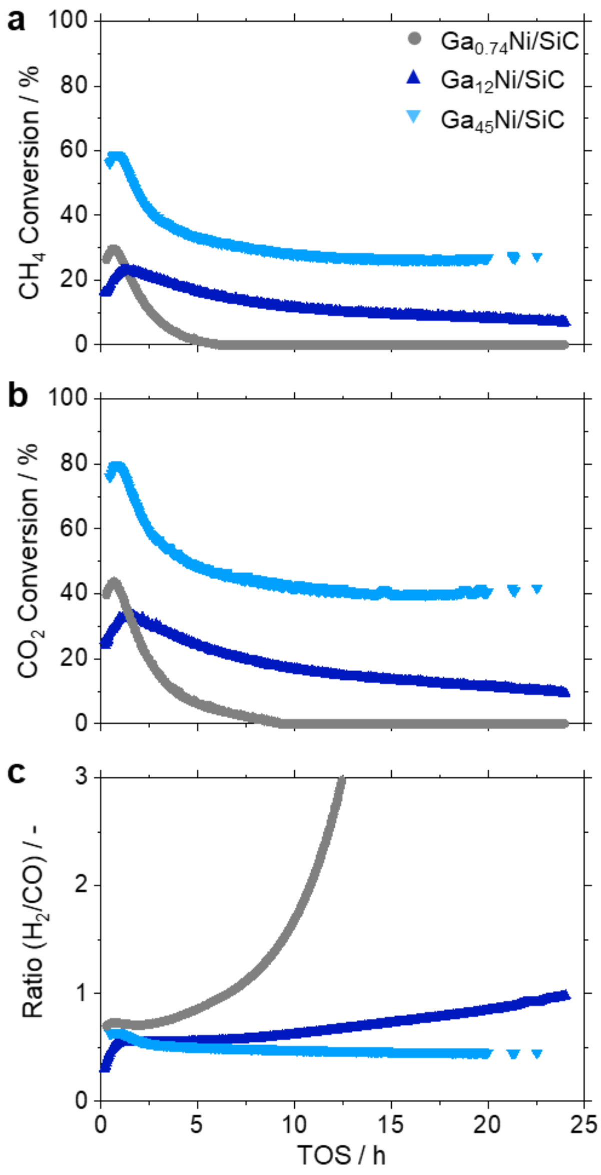

Figure S20: Conversion of (a) CH_4 and (b) CO_2, as well as (c) the obtained H_2/CO ratio in the product gas during dry reforming of methane over Ga-Ni SCALMS and a bimetallic reference catalyst with different concentrations of the active metal Ni employing a mesoporous SiC support. Metal loadings: 4.52 wt.% Ga and 0.08 wt.% Ni for $Ga_{45}Ni$/SiC, 5.44 wt.% Ga and 0.38 wt.% Ni for $Ga_{12}Ni$/SiC, 0.84 wt.% Ga and 0.72 wt.% Ni for $Ga_{0.74}Ni$/SiC. Reaction conditions: 900 °C, 1 bar, 1 g SCALMS, CH_4:CO_2:Ar = 1:1:3, 3 L_N g_{cat}^{-1} h^{-1}.

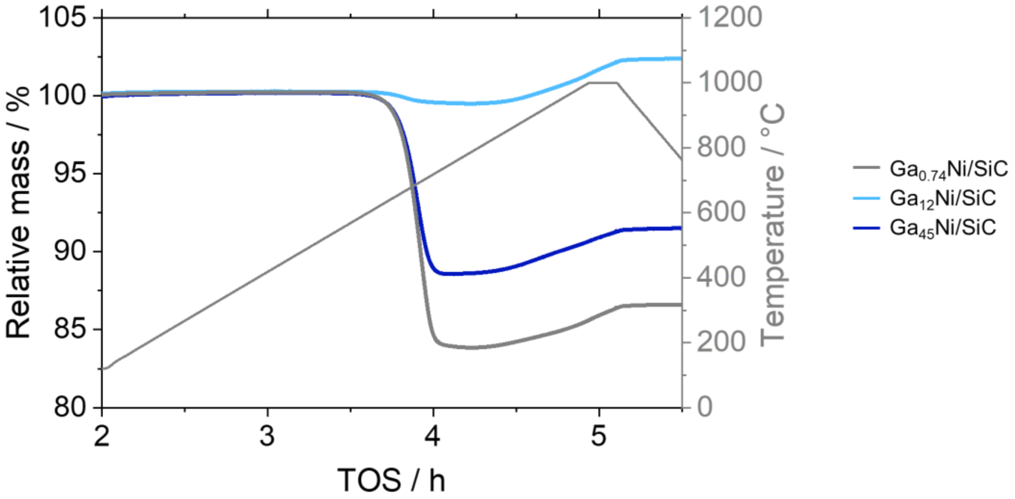

Figure S21: Thermogravimetric analysis during temperature-programmed oxidation of the spent Ga-Ni SCALMS and bimetallic reference catalyst with various ratios of gallium to nickel employing a mesoporous SiC support after application in dry reforming of methane.

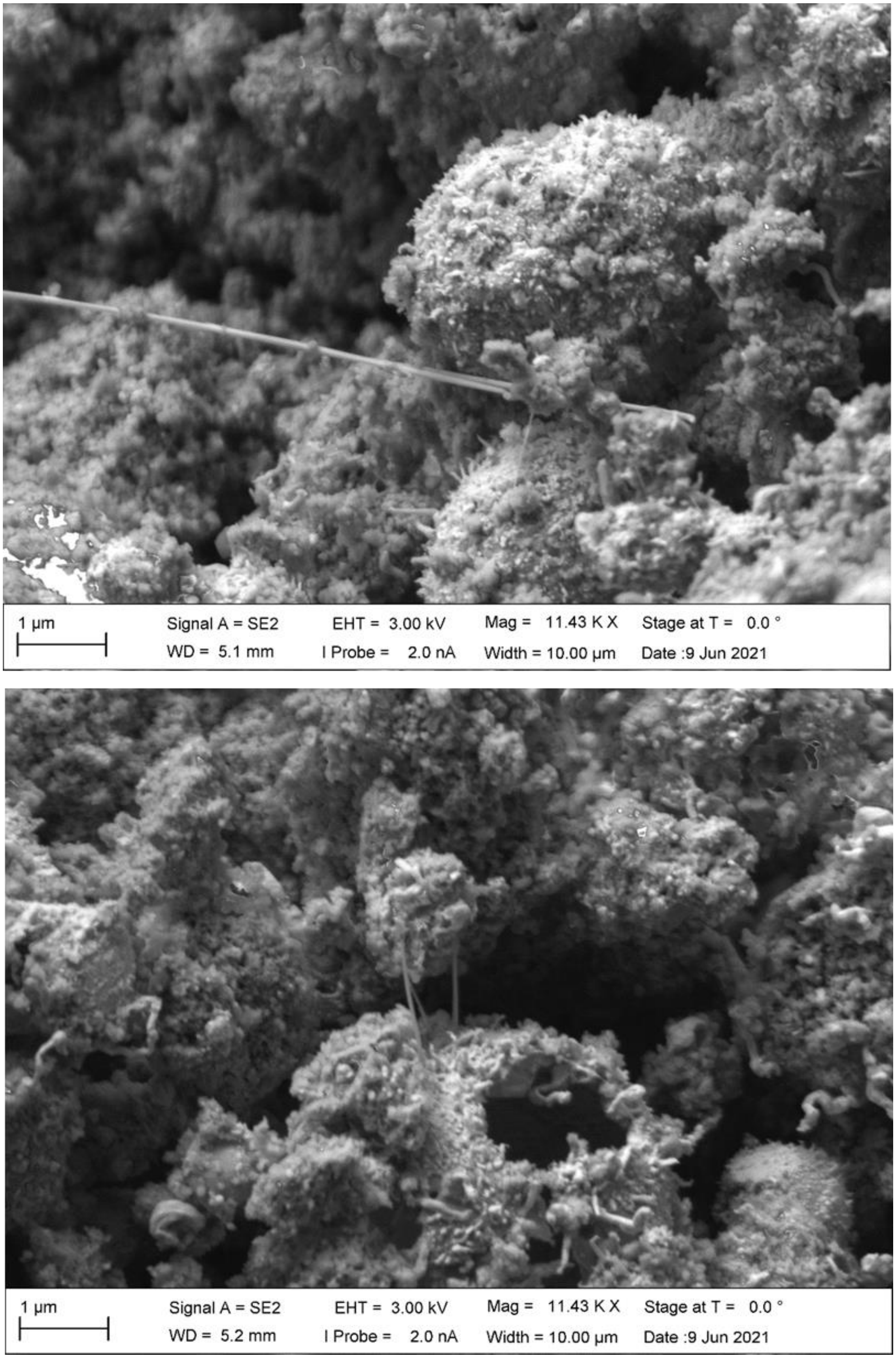

Figure S22: Scanning electron micrographs of $Ga_{12}Ni/SiC$ after catalytic application in DRM for 24 h with metal loadings of Ga and Ni of 5.44 wt.% Ga and 0.38 wt.%, respectively.

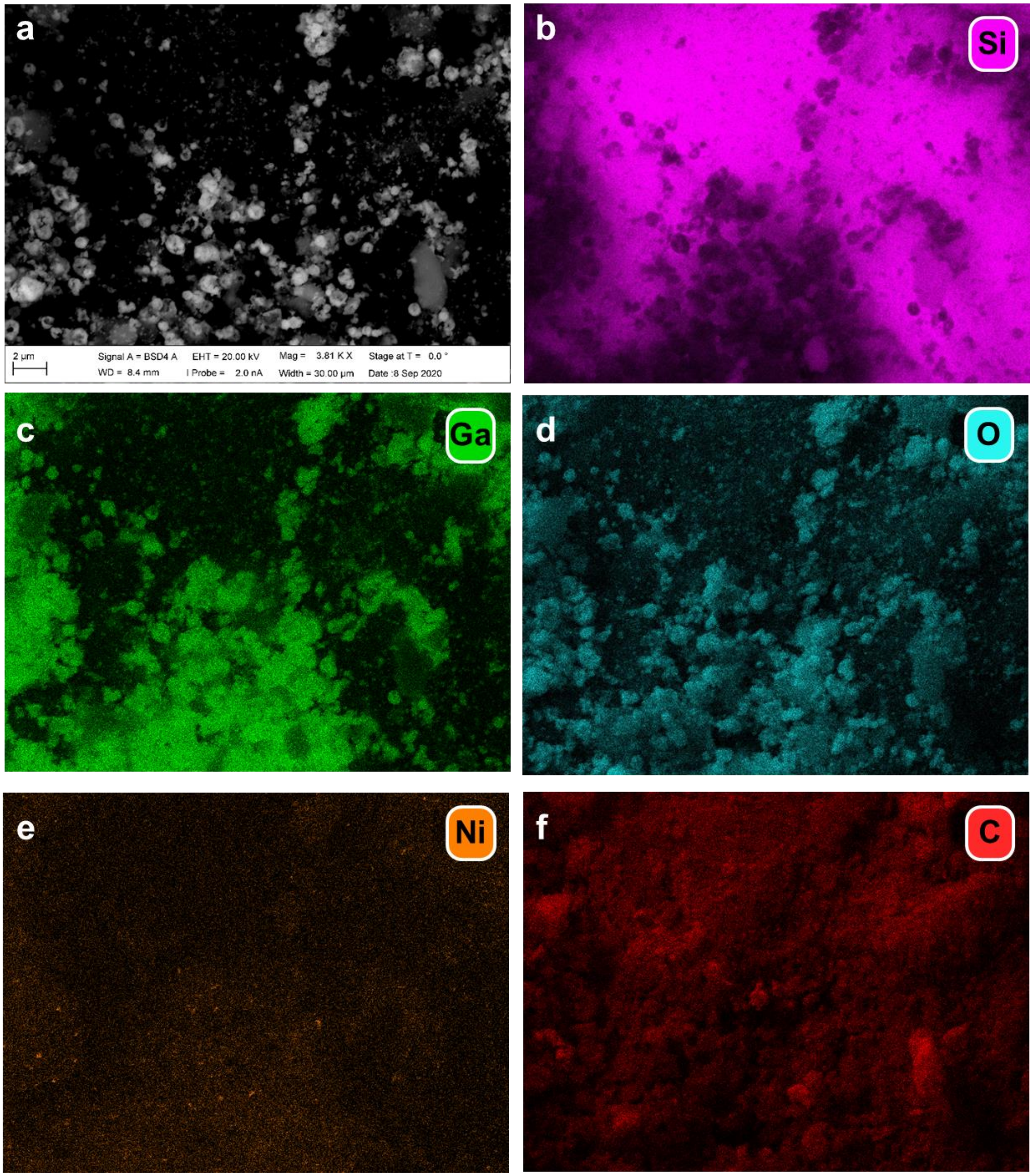

Figure S23: (a) Backscattered scanning electron micrograph with the corresponding (b-f) elemental maps via energy-dispersive X-ray spectroscopy of $Ga_{45}Ni/SiC$ after catalytic application in DRM for 24 h with metal loadings of Ga and Ni of 4.52 and 0.08 wt.%, respectively.

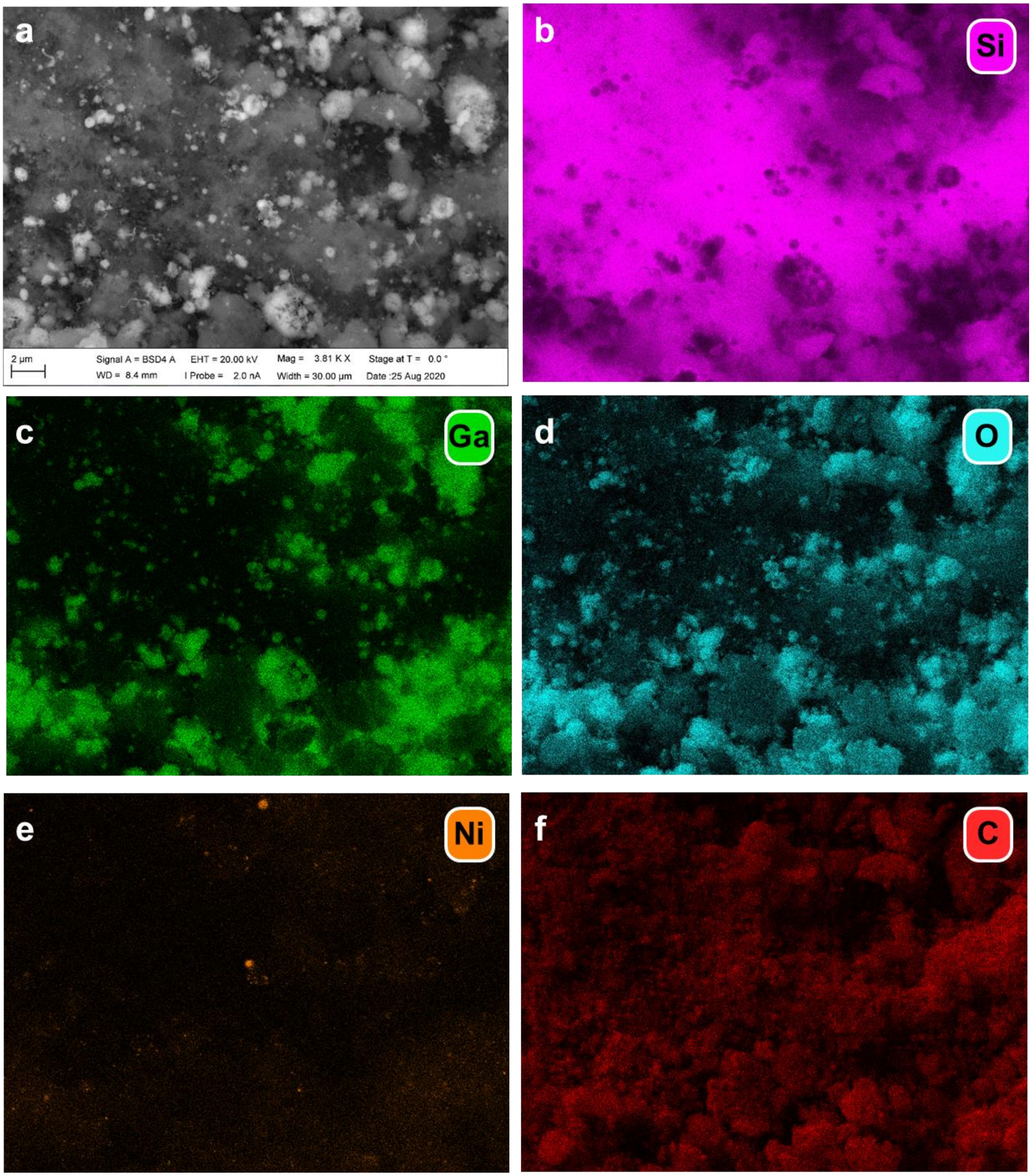

Figure S24: (a) Backscattered scanning electron micrograph with the corresponding (b-f) elemental maps via energy-dispersive X-ray spectroscopy of $Ga_{12}Ni/SiC$ after catalytic application in DRM for 24 h with metal loadings of Ga and Ni of 5.44 wt.% Ga and 0.38 wt.%, respectively.

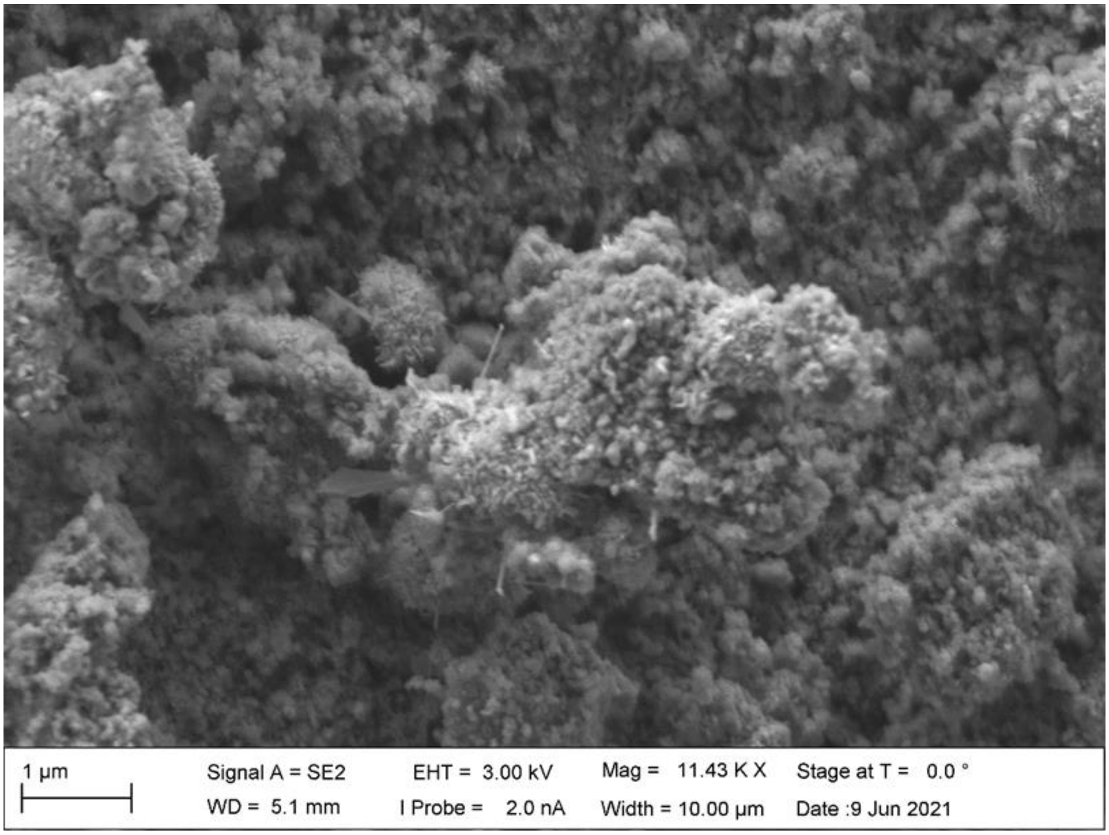

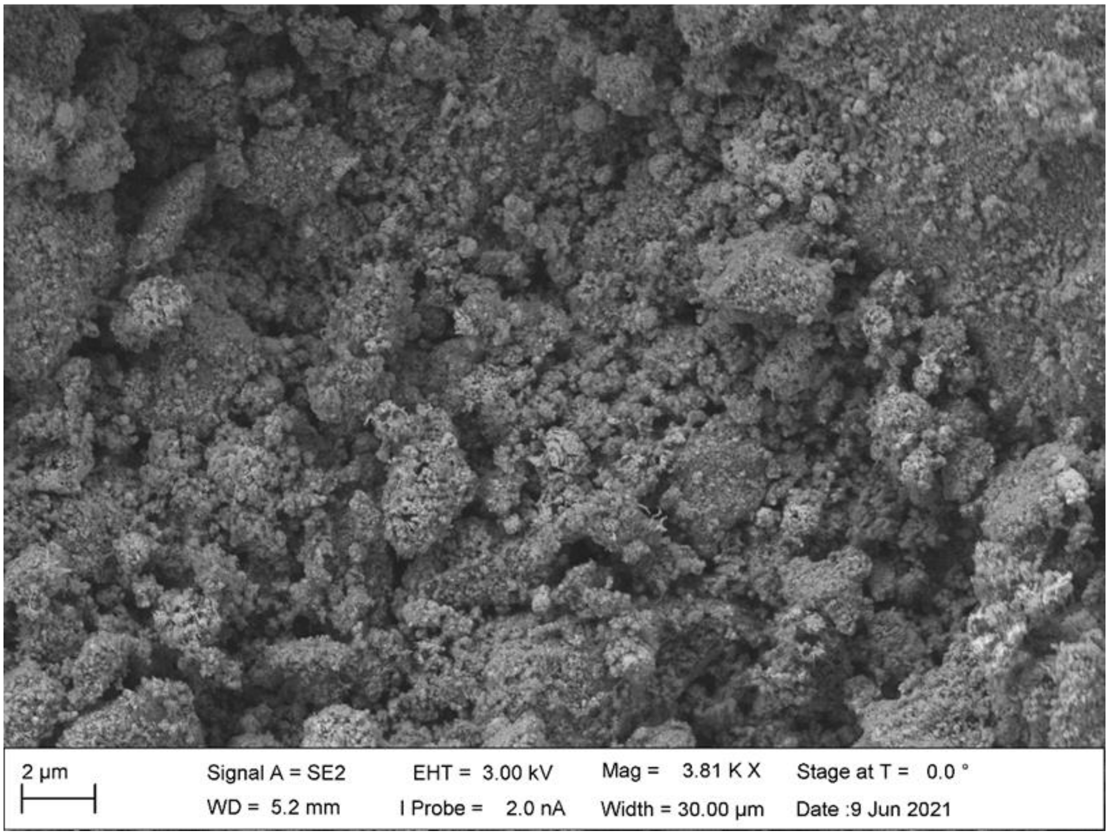

Figure S25: Scanning electron micrographs of $Ga_{45}Ni/SiC$ after catalytic application in DRM for 100 h with metal loadings of Ga and Ni of 4.52 and 0.08 wt.%, respectively.

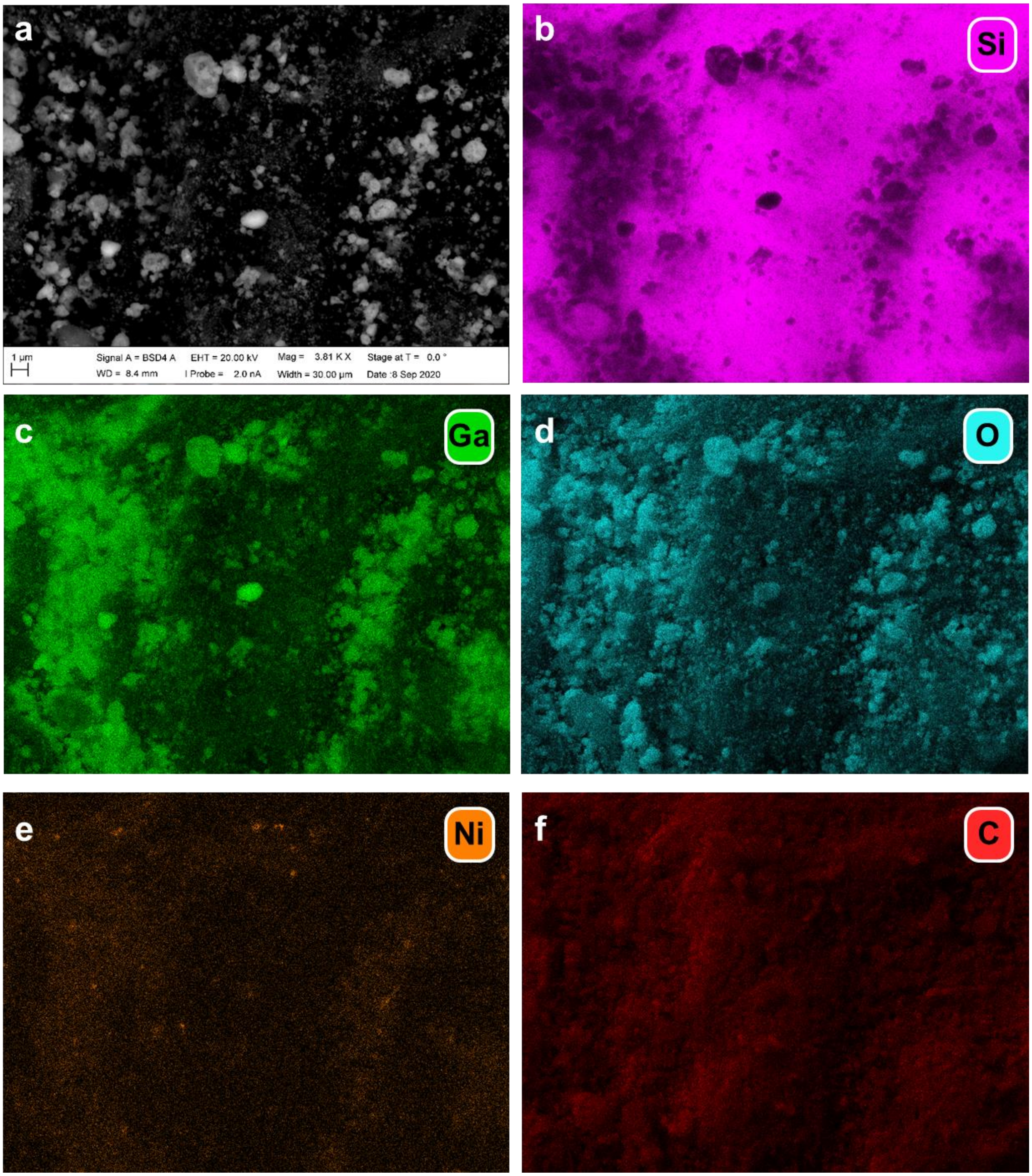

Figure S26: (a) Backscattered scanning electron micrograph with the corresponding (b-f) elemental maps via energy-dispersive X-ray spectroscopy of $Ga_{45}Ni/SiC$ after catalytic application in DRM for 100 h with metal loadings of Ga and Ni of 4.52 and 0.08 wt.%, respectively.

Supporting tables

Table S1: Intensity ratios of the D band over G band in Raman spectra of as prepared and spent SCALMS.

Catalyst	As prepared	Spent
$Ga_{43}Co/SiC$	1.10	1.29
$Ga_{85}Cu/SiC$	1.00	1.31
$Ga_{38}Fe/SiC$	1.08	1.32
$Ga_{45}Ni/SiC$	1.07	1.36
$Ga_{12}Ni/SiC$	1.07	1.16
SiC	1.03	1.45

Table S2: Chemical composition of the reference materials prepared and after application in dry reforming of methane.

Catalyst	Metal loading (as prepared) / wt.%		Molar ratio	Metal loading (spent)[a] / wt.%	
	Ga	Secondary metal		Ga	Secondary metal
Ga/SiC	5.50	-	-	4.06	-
$Ga_{0.74}Ni/SiC$	0.84	0.72	0.74	0.84	0.85

[a]Loadings of the spent catalysts were corrected for the amount of carbon as identified by means of TGA.

Supporting references

1 Barin, I., Knacke, O. & Kubaschewski, O. *Thermochemical properties of inorganic substances*. 1 edn, (Springer, 1973).

2 Barin, I., Knacke, O. & Kubaschewski, O. *Thermochemical properties of inorganic substances -- Supplement*. 1 edn, (Springer, 1977).

3 Knacke, O., Kubaschewski, O. & Hesselmann, K. *Thermochemical properties of inorganic substances*. 2 edn, (Springer, 1991).
